# Top stories on intracellular potassium channels in cardiac arrhythmia

**DOI:** 10.1016/j.hrthm.2024.07.012

**Published:** 2024-10

**Authors:** Harpreet Singh, Shridhar Kiran Sanghvi

**Affiliations:** 1 Department of Physiology and Cell Biology, The Ohio State University, Columbus, Ohio; 2 Department of Molecular, Cellular and Developmental Biology, The Ohio State University, Columbus, Ohio.

The intracellular K^+^ (_i_K^+^) channel translocation to the sarcolemma is linked with sinoatrial node (SAN) function and arrhythmic risk. Yet, the role of _i_K^+^ channels is poorly understood in atrial fibrillation (AF). In this article, we provide newer insights on _i_K^+^ channels to enhance our understanding of how their regulation contributes to arrhythmia.

## Spatially resolved multiomics of K^+^ channel in cardiac cells

K^+^ channels contribute to membrane repolarization and modulate the firing rate of the SAN cells. To maintain adequate [K^+^], _i_K^+^ stored in organelles and modulated by _i_K^+^ channels serves as a reservoir.^1^ In fact, cardiac cells accumulate a substantial amount of K^+^ during acute load. A study by Kanemaru and associates,^2^ through multimodal profiling of human hearts, described ion channels of the cardiac conduction system, where hyperpolarization-activated cyclic nucleotide–gated pacemaker channel genes (*HCN1* and *HCN4*) were enriched in pacemaker cells. *KCNJ5* and G protein–coupled receptors were enriched in the pacemaker cells along with SK2 (*KCNN2*) and THIK-1 (*KCNK13*) in nodal cells compared with atrioventricular bundle cells. BK_Ca_ (*KCNMA1*) and K_ATP_ (*KCNJ11*) are also reported in pacemaker cells. Hence, pathogenic mutations in these genes are implicated in SAN dysfunction and arrhythmias.

## BK_Ca_ channel in arrhythmia index

In excitable cells, the STREX isoform of BK_Ca_ localizes to the plasma membrane. The channel is formed by 4 poreforming a subunits and regulatory (β and γ) subunits. However, in adult cardiomyocytes, the DEC splice variant of BK_Ca_ targets the channel to the mitochondria.^1^ Pineda and coworkers^3^ identified and characterized the function of BK_Ca_ in SAN cells, which is associated with AF in aging humans. In human atrial appendages, the increased expression of STREX transcripts was observed with age, whereas the DEC and STREX transcripts increased within aging AF patients. The channel was reported in sarcoplasmic reticulum, in mitochondria, and in the vicinity of intercalate disks. Inactivation of BK_Ca_ showed no changes in normal cardiac pathophysiology; however, under β-adrenergic stimulation, the inhibition of the BK_Ca_ reduced the Ca^2+^ transient frequency. Also, a novel pathologic variant of BK_Ca_, p.S11_S12delinsG, disrupts the association of BK_Ca_ with β-subunit, which could explain the permanent AF in the probands.^3^ Overall, this study implicates BK_Ca_ in sinus node pathophysiology and atrial arrhythmia predisposition.

## K_ATP_ channel in atrial tachyarrhythmia

K_ATP_ channels are expressed in the heart and play a vital role in ischemic injury and AF. K_ATP_ comprises 4 pore-forming subunits, Kir6.1 and Kir6.2, and 4 regulatory subunits. In addition to the plasma membrane, K_ATP_ also localizes to intracellular organelles, which are implicated in K^+^ fluxes.^1^ Specifically, mitochondrial K_ATP_ is presumed to be encoded by SUR2/Kir6.2. Alternative splicing and expression of subunits can retain K_ATP_ in intracellular organellar membranes. The contribution of each pore-forming subunit in the hypoxia-induced atrial arrhythmia was teased apart by Specterman and colleagues.^4^ This study explored the electrophysiologic outcomes of K_ATP_ modulation and the contribution of each pore through direct comparison of global knockout mice by deleting K_ATP_ pore-forming subunits. Interestingly, the study showed that activating K_ATP_ during hypoxia maintains atrial effective refractory period in the heart that depends on Kir6.2. Moreover, knocking out Kir6.2 prevented hypoxia-induced atrial wavefront path-length and diminished induced atrial arrhythmias. In contrast, in Kir6.1^−/−^ mice, critical wavefront path-length shortening was observed with increased susceptibility to atrial arrhythmias during hypoxia. Overall, specific knockout of the pore-forming regions did modify electrophysiologic properties in the atrium but showed contrasting effects on preventing hypoxia-induced arrhythmias.^4^

## SK channel in AF

A study by Heijman and coworkers^5^ showed increase in SK currents resulting in the shortening of atrial refractory period in the atrial cardiomyocytes of patients with long-persistent chronic AF (cAF). The increase in the SK current is attributed to enhanced SK2 translocation to the sarcolemma in atrial cardiomyocytes. Furthermore, this translocation is promoted by an increase in protein phosphatase 2A, which decreases the phosphorylation of calmodulin-Thr80. Moreover, the in vitro experiments with tachypacing highlighted the importance of trans-sarcolemma Ca^2+^ influx on activating the SK current. This potential mechanism of increased SK2 activity could contribute to the rapid repolarization of atrial cardiomyocytes observed in cAF patients. This study provides mechanistic insight into the posttranslational regulation affecting the translocation of the membrane trafficking and the channel activity of SK in cAF patients. In addition to the sarcolemma, SK2 and SK3 channels are also found in inner mitochondrial membranes of cardiomyocytes, playing a vital role in protecting mitochondrial function and Ca^2+^ levels. SK2 channels in intracellular organelles can also contribute to repolarization of atrial cardiomyocytes.^5^

## Summary

Similar to intracellular Ca^2+^ levels, _i_K^+^ levels are tightly regulated by both plasma membrane and organellar K^+^ channels. In addition, _i_K^+^ levels also modulate ionic homeostasis in the cytosol. The major challenge in the _i_K^+^ channel field is to delineate the contribution of plasma membrane vs _i_K^+^, with the identification of organellar K^+^ channels being the first step.

## References

[1] Szabo I, Szewczyk A. Mitochondrial ion channels. Annu Rev Biophys 2023; 52:229–254. 37159294 10.1146/annurev-biophys-092622-094853

[2] Kanemaru K, Cranley J, Muraro D, Spatially resolved multiomics of human cardiac niches. Nature 2023;619:801–810. 37438528 10.1038/s41586-023-06311-1 PMC10371870

[3] Pineda S, Nikolova-Krstevski V, Leimena C, Conserved role of the large conductance calcium-activated potassium channel, K_Ca_1.1, in sinus node function and arrhythmia risk. Circ Genom Precis Med 2021;14:e003144. 33629867 10.1161/CIRCGEN.120.003144 PMC8058291

[4] Specterman MJ, Aziz Q, Li Y, Anderson NA, Hypoxia promotes atrial tachyarrhythmias via opening of ATP-sensitive potassium channels. Circ Arrhythm Electrophysiol 2023;16:e011870. 37646176 10.1161/CIRCEP.123.011870 PMC10510820

[5] Heijman J, Zhou X, Morotti S, Enhanced Ca^2+^-dependent SK-channel gating and membrane trafficking in human atrial fibrillation. Circ Res 2023; 132:e116–e133. 36927079 10.1161/CIRCRESAHA.122.321858 PMC10147588

